# Promoting teaching innovation among university teachers through AI literacy from the perspective of planned behavior: the moderating effects of three perceived supports

**DOI:** 10.3389/fpsyg.2025.1699174

**Published:** 2025-10-20

**Authors:** Yuping Zhao, Ling Huang

**Affiliations:** ^1^General Education Center, Communication University of China, Beijing, China; ^2^School of Business and Tourism Management, Postdoctoral Research Station in Business Administration, Yunnan University, Kunming, China

**Keywords:** Artificial Intelligence Literacy, teaching innovation, Theory of Planned Behavior, perceived support, university teachers

## Abstract

**Introduction:**

The rapid development of artificial intelligence (AI) is transforming higher education, yet the mechanisms through which AI literacy influences teaching innovation among university teachers remain insufficiently explored.

**Methods:**

This study, grounded in the Theory of Planned Behavior (TPB), investigates how AI literacy promotes teaching innovation via three psychological mechanisms: behavioral attitude, subjective norm, and perceived behavioral control. Additionally, the moderating effects of perceived support factors—teaching resources, peer support, and teaching autonomy—on the relationship between AI literacy and teaching innovation are considered. Empirical survey data from Chinese university teachers were used for analysis.

**Results:**

The findings reveal that AI literacy significantly enhances teachers’ behavioral attitudes, subjective norms, and perceived behavioral control, which in turn foster teaching innovation. Among these, perceived behavioral control plays the most significant role in driving innovative behavior. Moreover, teaching resources and teaching autonomy positively moderate the relationship between AI literacy and teaching innovation, while peer support only significantly influences behavioral attitudes.

**Discussion:**

These results extend the application of the Theory of Planned Behavior by uncovering the psychological mechanisms through which AI literacy fosters teaching innovation. The study provides empirical evidence supporting AI literacy training and teacher support in higher education, with implications for fostering innovation in teaching practices.

## 1 Introduction

The rapid development of artificial intelligence (AI) is profoundly transforming the ecosystem of higher education (Zhang, 2023). The widespread adoption of tools such as natural language processing, big data analysis, intelligent recommendations, and virtual teaching assistants is continuously reshaping university teachers’ daily teaching practices (Niloy et al., 2025). AI has been integrated into course design, learning analytics, and educational assessment, further expanding into classroom interactions, personalized learning support, and academic monitoring (Hwang et al., 2020). The teaching model is gradually shifting from teacher-centered to learner-centered, a fundamental change driven by technological advancements (Sperling et al., 2024). This shift is part of a broader “educational paradigm transformation,” where the focus moves toward more student-centered, personalized learning experiences, significantly improving teaching efficiency and flexibility (Wang et al., 2025). With the increasing application of AI technologies, university teachers are facing growing demands to update their skills and redefine their roles, shifting from traditional “knowledge transmitters” to “learning guides” and “innovative practitioners” (Kim, 2025).

Teaching innovation, as one of the key responsibilities of university teachers, is a core manifestation of professional development and a necessary condition for modernizing education and cultivating innovative talent in higher education (Wang et al., 2025). It involves the continuous exploration and improvement of teachers’ educational philosophies, course goals, teaching methods, and assessment practices (Gerçek and Özveren, 2025). In this context, AI literacy has become a key factor for teachers to adapt to educational transformation and foster teaching innovation. AI literacy is generally defined as an individual’s ability to understand, apply, and critically reflect on AI (Kelley and Wenzel, 2025; Ozudogru and Durak, 2025). For university teachers, it not only encompasses technical operations and tool applications but also includes the ability to evaluate educational values, identify potential risks, and creatively integrate AI into teaching (Ji et al., 2025).

Existing studies suggest that AI literacy directly influences classroom effectiveness, student experiences, and teachers’ professional development (Liu et al., 2025). Teachers with higher AI literacy are more likely to break away from traditional models and demonstrate greater innovation in course design and educational assessment (Guan et al., 2025). Most research has focused on performance outcomes and technology adoption, emphasizing the relationship between literacy and tool usage, technology acceptance, and efficiency, but has insufficiently explored how AI literacy influences teaching innovation through psychological and behavioral mechanisms (Duong, 2025). In contrast, studies on student AI literacy are more systematic, while research on teachers is relatively scarce (Tzirides et al., 2024).

Therefore, investigating the impact of AI literacy on teaching innovation among teachers is of significant theoretical and practical importance. Although some scholars have suggested that AI literacy may promote teaching innovation, the underlying mechanisms remain unclear, and the impact of psychological and social factors lacks systematic explanation (Zhou et al., 2025). Existing studies often rely on models such as the Technology Acceptance Model (TAM) or Unified Theory of Acceptance and Use of Technology (UTAUT), focusing on adoption intentions while neglecting the psychological processes and perceived support environments in teaching practice (Yang et al., 2025). It is essential to reconsider the relationship between AI literacy and teaching innovation from the perspectives of psychology and organizational behavior.

The Theory of Planned Behavior (TPB) provides a solid psychological framework for understanding teachers’ innovative behaviors (Zhang, 2025). According to this theory, an individual’s behavioral intentions are primarily determined by behavioral attitude, subjective norm, and perceived behavioral control (Dunn et al., 2018). AI literacy may influence teachers’ attitudes toward the educational value of AI, their perception of external expectations, and their confidence in their abilities, thereby promoting teaching innovation (Kong et al., 2024). At the same time, external supportive conditions cannot be overlooked in this process (Adabor et al., 2025). Perceived support theory posits that educational resources, peer support, and teaching autonomy enhance motivation and foster creative behaviors (Han et al., 2021). Educational resources provide material support for teachers to explore new methods, peer support stimulates motivation through collaborative exchange, and teaching autonomy creates institutional space for trying innovations (Cai and Tang, 2022; Hornstra et al., 2021; Nshimiyimana and Cartledge, 2020). Research has shown that a supportive environment can amplify the positive psychological and behavioral effects of an individual’s capabilities (Wang et al., 2025). Thus, external situational support may moderate the impact of AI literacy on teachers’ attitudes, subjective norms, and perceived control, indirectly influencing their level of teaching innovation.

In summary, existing research has the following limitations: First, there is limited systematic research on the relationship between AI literacy and teaching innovation, especially empirical studies focusing on university teachers (Chou et al., 2025); second, existing studies overly rely on technology adoption frameworks, lacking a comprehensive perspective that integrates psychology and organizational behavior (Sanusi et al., 2024); third, there is insufficient research on the role of external situational factors, such as educational resources, peer support, and teaching autonomy, in influencing teaching innovation (Ding et al., 2024; Mnguni et al., 2024).

Therefore, this study focuses on Chinese university teachers and attempts to construct and validate a comprehensive model to systematically explore how AI literacy influences teaching innovation through teachers’ cognitive and psychological processes. Furthermore, the study investigates the role of external factors such as teaching resources, peer support, and teaching autonomy. This study seeks to answer the following three core questions: (1) Does AI literacy significantly promote teaching innovation among university teachers? (2) What role do behavioral attitude, subjective norm, and perceived behavioral control play in this process? (3) Does the supportive environment strengthen or weaken the relationship between AI literacy and teaching innovation?

By answering these questions, this study will not only contribute to a deeper understanding of the relationship between AI literacy and teaching innovation among university teachers but also provide empirical support and practical insights for the digital transformation of education and the professional development of teachers. The subsequent sections of the paper will present the theoretical foundation, literature review, research model and hypotheses, research methods, data analysis and results, and discussion and conclusion.

## 2 Theoretical framework and literature review

### 2.1 Theory of Planned Behavior

The Theory of Planned Behavior (TPB) posits that an individual’s behavioral intentions are primarily determined by three psychological factors: behavioral attitude, subjective norm, and perceived behavioral control (Ajzen, 1991). Behavioral attitude refers to an individual’s positive or negative evaluation of the likely outcomes of a specific behavior (Ahadzadeh et al., 2024). Subjective norm reflects an individual’s perception of the expectations and social pressures from others in a given social context (Thanki et al., 2024). Perceived behavioral control represents an individual’s assessment of their resources and abilities; the more an individual believes they have the necessary conditions and fewer potential barriers, the stronger their behavioral intention will be (Ateş, 2020). Perceived behavioral control is an internal psychological factor that determines a person’s belief in their ability to perform a behavior successfully. It refers to teachers’self-efficacy, or their confidence in overcoming challenges and utilizing their abilities to incorporate AI tools into their teaching practices (Hamm et al., 2024). These three factors interact and collectively explain the formation of behavioral intentions and their translation into actual behaviors (Hou et al., 2022).

In educational research, TPB has been widely applied, particularly in explaining teachers’ teaching behaviors and technology adoption (Frawley and Campbell, 2025). Studies have shown that positive behavioral attitudes enhance teachers’ willingness to engage in curriculum reform and adopt new tools. External expectations and pressures, such as school policies, peer support, and student feedback, influence teaching choices through subjective norms. Teachers’ confidence in their abilities and external conditions, known as perceived behavioral control, ultimately determines whether behavioral intentions translate into actual actions (Andersen et al., 2019; Gold et al., 2024).

As AI gradually integrates into higher education, teachers’ AI literacy may influence all three dimensions of TPB, shaping their attitudes toward the educational value of AI, enhancing their sensitivity to social norms, and strengthening their self-efficacy (Ahadzadeh et al., 2024). Therefore, TPB provides an important theoretical framework for understanding how university teachers can achieve teaching innovation through AI-driven processes.

### 2.2 AI literacy

AI literacy is initially defined as an individual’s ability to understand, use, and evaluate AI systems (Almatrafi et al., 2024). Its core includes not only knowledge of AI principles and mechanisms but also the ability to use AI tools effectively in real-world contexts and critically reflect on their social, ethical, and educational impacts (Ng et al., 2021). Compared to information and digital literacy, AI literacy places greater emphasis on algorithmic thinking and human-machine collaboration, and is regarded as an interdisciplinary and cross-contextual competency (Senoner et al., 2024).

As AI becomes more deeply applied in education, the concept of AI literacy has evolved from early tool-based operation to a broader competency that includes technological integration, interdisciplinary collaboration, ethical judgment, and social responsibility (Sperling et al., 2024). For teachers, AI literacy is both a prerequisite for the digital transformation of education and a critical driver of teaching innovation (Ozudogru and Durak, 2025). High levels of AI literacy can not only help teachers develop positive attitudes toward technology adoption but also reduce anxiety caused by technological uncertainty, enabling greater flexibility and creativity in course design and classroom management (Hwang and Wu, 2025).

Existing studies have identified the multidimensional characteristics of AI literacy. One stream of research emphasizes its ethical and critical dimensions, suggesting that individuals should be able to assess AI outputs and potential risks in different contexts (Kelley and Wenzel, 2025; Ozudogru and Durak, 2025). Another stream highlights the interactive dimension, noting that AI literacy involves not only cognitive and operational skills but also the ability to interact and collaborate with intelligent systems (Ayanwale et al., 2024). In the professional development of teachers, AI literacy integrates technical knowledge, teaching skills, and ethical judgment to support teachers in making informed decisions in complex educational settings (Al-Mughairi and Bhaskar, 2024; Ning et al., 2025). Overall, AI literacy for teachers is defined as a systemic competency that encompasses technical operation, algorithmic thinking, interdisciplinary integration, and social impact assessment (Abulibdeh et al., 2024; Bewersdorff et al., 2025).

**Figure 1:**
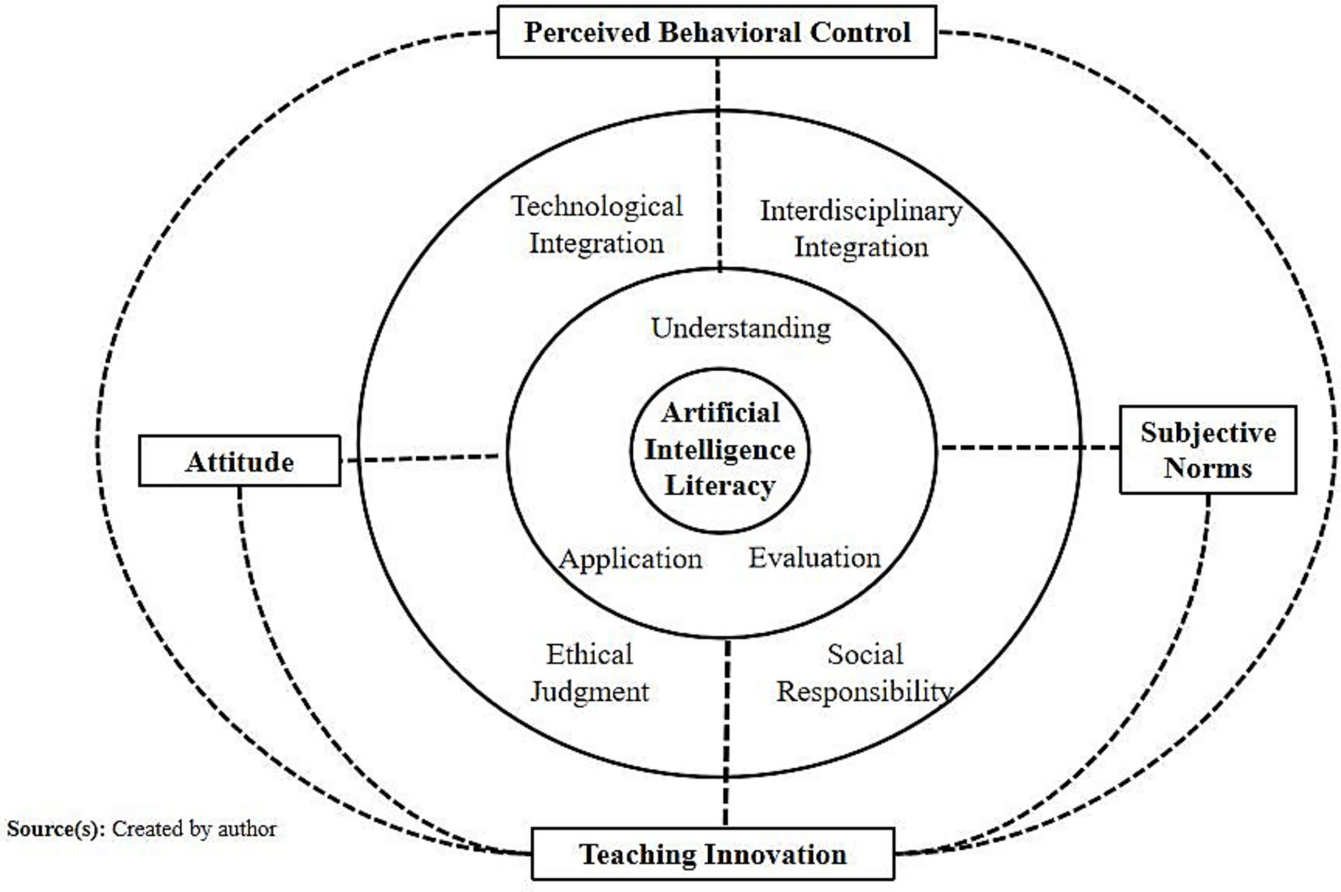
Evolution of AI literacy and the framework for teacher teaching innovation.

Empirical studies have further validated the relationship between AI literacy and teaching innovation. Research indicates that AI literacy can not only directly promote innovative practices by enhancing teachers’ technical proficiency but also indirectly foster innovation by shaping positive cognitive attitudes, strengthening the perception of social expectations, and boosting self-efficacy (Ivanov et al., 2024). Table 1 systematically reviews the latest research on AI and digital technologies in teaching innovation, providing a solid foundation for the construction of this study’s model. Building on this, Figure 1 presents the evolution and application framework of AI literacy: its core is composed of the initial definitions (understanding, application, evaluation), with extensions to dimensions such as technological integration, interdisciplinary collaboration, ethical judgment, and social responsibility. These literacy components influence teaching innovation through the three psychological mechanisms of TPB—attitudes, subjective norms, and perceived behavioral control—forming a logical chain of “AI Literacy—TPB Psychological Mechanisms—Teaching Innovation” that lays a systematic foundation for the theoretical model of this study.

**Table 1:** Research progress on AI and digital technologies in teaching innovation.

Reference	Research context	Research method	Research finding
Panday-Shukla (2025)	AI literacy	Quantitative Research	Pre-service teachers and teacher educators have moderate digital literacy but low AI literacy.
Ozudogru and Durak (2025)	Artificial Intelligence	Quantitative Research	AI readiness (cognition, vision, and ethics) significantly impacts AI-enhanced innovation levels in teaching.
Chen et al. (2025)	AI Technologies	Quantitative Research	Adequate technical support and adaptable AI tools are crucial for integrating AI into STEM education.
Chu and Wang (2025)	AI-Integrated	Quantitative Research	Micro and individual factors, especially beliefs in AI’s potential, significantly impact teachers’ epistemic agency, fostering innovation.
Chen and Zou (2024)	Intelligent MR devices	Quantitative Research	Intelligent teaching devices enhance educational equity and teaching quality, particularly in remote areas.
Robayo-Pinzon et al. (2024)	Artificial Intelligence	Quantitative Research	Students generally agree with the co-creation of value through AI functions in higher education scenarios.
Lin and Chen (2024)	Artificial intelligence	Quantitative Research	AI applications can constrain creativity and innovation due to rigid frameworks.
Kim (2024)	Artificial intelligence	Quantitative Research	Optimizing the complementary strengths of both human teachers and AI holds great potential for educational innovation.

Source(s): created by author.

### 2.3 Teaching innovation

Teaching innovation is generally understood as the process through which teachers introduce new ideas and tools into their teaching philosophies, methods, and practices to improve learning outcomes and the teaching environment (Gilbert et al., 2021). It involves not only the adoption and integration of classroom technologies but also the transformation of course design, assessment methods, and teacher-student interaction patterns (López et al., 2023). In higher education, teaching innovation is characterized by the selection of diverse methods, integration of interdisciplinary resources, and personalized responses to learners’ needs, making teaching more flexible, open, and adaptive (Miranda et al., 2021).

Existing research generally agrees that teaching innovation is influenced by both individual and contextual factors (Kottmann et al., 2024). On the individual level, a teacher’s knowledge structure, innovation awareness, and technical abilities determine the likelihood of implementing changes in their teaching (Chen, 2024). On the contextual level, institutional support, peer collaboration, and technological environments have been identified as key conditions for promoting teaching innovation (Mokhlis and Abdullah, 2024). In recent years, the widespread application of AI has further expanded the boundaries of teaching innovation. It not only provides tools like learning analytics and intelligent feedback but also facilitates the paradigm shift from a “teacher-centered” to a “learner-centered” model (Chou et al., 2025). However, teachers still face challenges in advancing innovation, including insufficient AI literacy, limited teaching autonomy, and uncertainty about new teaching models (Gupta and Bhaskar, 2020). Table 2 summarizes the latest research on teaching innovation, covering the research context, methods, and key findings. These results indicate that teacher innovation relies both on individual cognition and attitudes and on the important influence of external support environments. This lays the foundation for exploring the “AI literacy—TPB psychological mechanisms—teaching innovation” pathway in this study.

**Table 2:** Overview of research progress on teaching innovation.

Reference	Research context	Research method	Research finding
Xiang et al. (2024)	Career calling	Quantitative Research	Career calling is positively correlated with teacher innovation.
Cai and Tang (2021)	School support	Quantitative Research	The impact of school support for innovation on teacher innovation varies.
Liu et al. (2024)	Conceptualizations	Qualitative Research	Domain-specific definitions aid in understanding teacher innovation.
Liu et al. (2022)	Professional learning communities	Quantitative Research	School-level professional learning communities positively influence individual teacher innovation.
Han et al. (2021)	Perceived support	Qualitative Research	The relationship between teaching resources and teacher innovation is minimal.
Teng et al. (2024)	Distributed leadership	Qualitative Research	Distributed leadership impacts teacher innovation at both team and individual levels.
Ertas and Pekmezci (2025)	Career motivation	Qualitative Research	Instructional practice and teacher innovation mediate the relationship between social utility motivation and job satisfaction.
Bao (2024)	Principals’ secure base leadership	Qualitative Research	Principals’ secure base leadership enhances teacher innovation through affective commitment.
Qin et al. (2025)	Teacher collaboration	Qualitative Research	Teacher collaboration significantly boosts innovation ability and teaching motivation.
Ma and Zhang (2025)	Distributed leadership	Qualitative Research	Distributed leadership does not directly predict teacher innovation behavior.
Adams et al. (2025)	Openness to experience	Qualitative Research	Teachers’ openness to experience significantly predicts creativity, LMX quality, and innovative teaching practices.

Source(s): created by author.

### 2.4 Perceived support

Perceived support refers to an individual’s subjective perception of the available resources, social relationships, and autonomy within an organizational context. It is widely recognized as a critical psychosocial factor influencing motivation, behavior, and innovation (Wahid and Ayub, 2024). In the context of higher education, perceived support for teachers not only stems from institutional guarantees and material resources but also includes emotional recognition and social support derived from peer collaboration, organizational atmosphere, and management mechanisms (Cai and Tang, 2022). These elements help to stimulate positive teaching attitudes and enhance innovative motivation (Liu and Chang, 2023).

In the context of university teachers adapting to and applying AI technologies in their teaching practices, perceived support can be broken down into three key dimensions: teaching resources, peer support, and teaching autonomy (Kruse et al., 2024). These three dimensions are distinct but interrelated, and together they provide a comprehensive framework of support that enables teachers to navigate the challenges of AI integration in teaching.

Teaching resources, such as AI training opportunities, access to digital tools, and platform infrastructure, provides the technological foundation for teachers to improve their AI literacy and drive classroom innovation (Padilha et al., 2021). High levels of resource support enhance teachers’ understanding and control over AI tools, lowering the barriers to technology adoption and increasing their willingness to actively incorporate AI into their teaching (Cai and Tang, 2022).

Peer support plays a buffering and motivating role in the adoption of AI technologies (Hornstra et al., 2021). Collaboration and communication among teachers not only help share experiences of AI teaching practices and reduce the uncertainty associated with technology, but also provide emotional support and a sense of belonging, thereby boosting teachers’ technological confidence and innovative motivation (Adie et al., 2024). This is especially important in the context of rapid AI tool iterations.

Teaching autonomy refers to the freedom and decision-making power that teachers have in course design, teaching methods, and the selection of teaching tools (Zhao and Qin, 2021). Teachers with higher levels of teaching autonomy are better able to independently adjust and innovate their teaching methods, particularly as AI tools become integrated into their teaching practices (Martinek et al., 2020). Teaching autonomy enhances teachers’ sense of ownership over AI integration, enabling them to adapt AI tools to better meet the needs of their students and teaching objectives (Vangrieken and Kyndt, 2020). It facilitates the transformation of technical competence into classroom practices and encourages teachers to adopt new, creative approaches in response to the dynamic educational landscape (Bali et al., 2025).

As AI continues to be embedded in educational practices, the diversity of educational resources, the continuity of peer support, and the enhancement of teaching autonomy provide critical psychological foundations for teachers to translate AI literacy into teaching innovation behaviors (Okada, 2023; Zhao et al., 2021). These three types of perceived support not only mitigate psychological barriers during the technology adoption process but also stimulate teachers’ sense of technological efficacy and autonomy, playing an irreplaceable role in moderating and empowering the integration of AI in teaching.

## 3 Research model and hypotheses development

### 3.1 AI literacy and the Theory of Planned Behavior (behavioral attitude, subjective norm, and perceived behavioral control)

AI literacy reflects a teacher’s comprehensive understanding of knowledge, operational skills, and critical thinking, which influences their acceptance and use of AI tools in teaching contexts (Ma and Lei, 2024). Teachers with higher AI literacy are more likely to recognize the potential of AI in enhancing classroom efficiency and improving learning experiences, gradually forming a positive attitude (Wang and Wang, 2024). Attitude is not simply an emotional preference but represents a deeply cognitive and value-based stance toward AI integration. Teachers with positive attitudes are more likely to engage with AI technologies, incorporating them into course design, classroom interactions, and assessment methods (To et al., 2023). This stable orientation provides the psychological momentum for teaching innovation, making innovative behaviors more common. AI literacy also influences teachers’ perceptions of external norms. In line with the Theory of Planned Behavior (TPB), teachers’ behaviors are significantly influenced by the subjective norms around them, such as policy support, disciplinary communities, and student expectations (Dierendonck et al., 2024). Teachers with higher AI literacy are more likely to internalize these external norms as part of their professional identity, strengthening their sense of responsibility and enhancing their innovative behavior (Adelana et al., 2024). Perceived behavioral control is similarly affected by AI literacy. AI literacy enhances teachers’ sense of self-efficacy, allowing them to manage classroom uncertainty, break down tasks, and remain confident even in the face of technical difficulties (Chen et al., 2023). This is particularly crucial under limited resources, as it reduces psychological resistance caused by uncertainty and increases teachers’ willingness to engage in innovative behaviors (Hamm et al., 2024). AI literacy shapes teachers’ psychological readiness and behavioral tendency for teaching innovation through the three dimensions of attitude, subjective norm, and perceived behavioral control. Based on this, the following hypotheses are proposed:

*H1a. AI literacy has a significant positive effect on teachers' behavioral attitude.*

*H1b. AI literacy has a significant positive effect on teachers' subjective norm.*

*H1c. AI literacy has a significant positive effect on teachers' perceived behavioral control.*

### 3.2 The Theory of Planned Behavior (behavioral attitude, subjective norm, and perceived behavioral control) and teaching innovation

In the teaching domain, the three components of the Theory of Planned Behavior form the key psychological foundation for teachers’ innovation. Behavioral attitude represents teachers’ cognition and emotional experience regarding the value of AI in teaching (Liu and Wang, 2024). Positive attitudes, informed by cognitive appraisals and emotional investments, are critical in fostering teachers’ willingness to experiment with new tools, restructure course plans, and engage in repeated trials, all of which enhance the scope and depth of innovation. As the attitude becomes more stable, teachers are more inclined to adopt structured and adaptive methods in course design, incorporating intelligent feedback, layered support, and data-driven evaluation, thus extending innovative practices (Ehlert et al., 2022). Subjective norm represents the societal and professional expectations placed on teachers. This dimension underscores the influence of external norms, such as policy guidelines, peer practices, and student demands, in shaping teachers’ professional responsibilities (Knauder and Koschmieder, 2019). Teachers’ internalization of these norms not only strengthens their social responsibility but also increases their commitment to adopting innovations, as external pressures and professional values align. Perceived behavioral control reflects teachers’ judgment of feasibility and control during the innovation process. Teachers with stronger control can break down complex goals into manageable tasks, maintain steady progress in resource-constrained situations, and use data feedback for continuous improvement (Zhan et al., 2024). Perceived behavioral control, as influenced by self-efficacy, determines how confidently teachers can face challenges, overcome failures, and persist in innovative efforts, transforming the innovation process from trial and error to a sustained, systematic practice. Attitude, norm, and control impact cognition, social aspects, and operations, respectively, collectively driving teachers to transform potential intentions into visible practices, forming an intrinsic motivation system for teaching innovation (Ateş, 2020). Based on this, the following hypotheses are proposed:

*H2a. Behavioral attitude has a significant positive effect on teachers' teaching innovation.*

*H2b. Subjective norm has a significant positive effect on teachers' teaching innovation.*

*H2c. Perceived behavioral control has a significant positive effect on teachers' teaching innovation.*

### 3.3 The moderating role of perceived support (teaching resources, peer support, teaching autonomy)

Teaching resources are key conditions for teachers to engage in innovative practices, encompassing hardware, software platforms, training opportunities, and institutional support (Wu et al., 2022). According to the Theory of Planned Behavior, environmental conditions significantly influence the formation of attitudes, norms, and perceived control (Wang et al., 2025). The availability of resources determines whether teachers can effectively translate their AI literacy into positive psychological mechanisms (Ateş, 2020). In teaching contexts, abundant resources provide both material and emotional support, helping to build teachers’ confidence in the application of AI tools. Regarding attitudes, abundant resources reduce the risks of practice, making it easier for teachers to translate literacy into positive evaluations. Equipment and services provide a safety net, creating value convictions and emotional investment during operations (Ayanwale et al., 2025). This material and emotional safety net helps solidify teachers’ commitment to AI integration and teaching innovation, reducing psychological barriers to innovation. Subjective norms also depend on resource support. Resources not only provide material conditions but also symbolize the organization’s and community’s focus on AI teaching, leading teachers to perceive stronger external recognition and expectations (Ramnarain et al., 2024). As resources grow, teachers perceive a stronger alignment with institutional and professional goals, reinforcing their commitment to innovation. Perceived behavioral control is more closely related to resources. Available tools and services give teachers more control in complex situations, enhancing self-efficacy and promoting the realization of innovation intentions (Gong, 2023). Resources act as “magnifiers.” While literacy provides knowledge and skills, the positive effects of literacy are hard to fully utilize in the absence of resources. When resources are sufficient, the positive effects of literacy on attitude, norms, and control are strengthened, making it easier for innovation motivation to be converted into action. The following hypotheses are proposed:

*H3a. Teaching resources positively moderate the relationship between AI literacy and behavioral attitude.*

*H3b. Teaching resources positively moderate the relationship between AI literacy and subjective norm.*

*H3c. Teaching resources positively moderate the relationship between AI literacy and perceived behavioral control.*

Peer support reflects the emotional encouragement, experience sharing, and role modeling teachers receive within teams and academic communities. Social support theory indicates that positive peer interactions can alleviate stress and enhance innovation confidence (Wu et al., 2022). From the perspective of the Theory of Planned Behavior, peer support, as an important aspect of the social environment, has a significant influence on the formation of attitudes, subjective norms, and perceived behavioral control (Frawley and Campbell, 2025). Peer support, through collaborative interactions and shared experiences, reduces the isolation teachers may face and amplifies the social and emotional aspects of innovation. On the attitude level, even if teachers possess AI literacy, without peer encouragement, it is difficult to transform cognitive advantages into emotional investment (Zhou et al., 2022). A positive team atmosphere and practical demonstrations help build confidence and positive emotions (Sokha, 2024). Subjective norms are strengthened by peer support. Compared to policy documents, the adoption and demonstration by colleagues are more persuasive, leading teachers to perceive group recognition and internalize it as professional responsibility (Zhao et al., 2024). The collective validation from peers helps solidify teachers’ understanding of their innovation efforts as valid and valuable within their professional community. Perceived behavioral control also benefits from peer support. Collaboration and mutual assistance prevent teachers from facing technical or teaching challenges in isolation, enhancing control and willingness to act (Wan et al., 2024). Peer support not only shares resources but also provides social validation. Teachers, in a group-acknowledged environment, feel the practical value of their efforts and are more likely to transform innovation into normalized behavior. Therefore, peer support can strengthen the effect of AI literacy on the elements of the Theory of Planned Behavior, turning it into genuine innovative motivation. The following hypotheses are proposed:

*H4a. Peer support positively moderates the relationship between AI literacy and behavioral attitude.*

*H4b. Peer support positively moderates the relationship between AI literacy and subjective norm.*

*H4c. Peer support positively moderates the relationship between AI literacy and perceived behavioral control.*

Teaching autonomy reflects the degree of freedom teachers have in course design, teaching methods, and tool selection. Self-determination theory emphasizes that autonomy can stimulate intrinsic motivation, increasing engagement and innovation willingness (Reeve and Cheon, 2024). In the framework of the Theory of Planned Behavior, autonomy is an important external condition affecting attitudes, norms, and perceived control, determining whether AI literacy can translate into positive psychological mechanisms (Ren, 2024). Autonomy provides teachers with a sense of ownership over their teaching, which in turn enhances the value they place on innovation and the integration of AI. On the attitude level, teachers with AI literacy, but limited in teaching activities, find it difficult to form positive emotions. As autonomy increases, teachers can freely apply AI tools based on their preferences, creating value convictions (Vangrieken and Kyndt, 2020). Subjective norms are more likely to internalize due to autonomy. Teachers can combine external requirements with personal will, shifting from passive compliance to professional recognition (Martinek et al., 2020). Perceived behavioral control also depends on autonomy. Greater freedom reduces external barriers, enhancing teachers’ sense of control and self-efficacy (Miao and Ma, 2023). By increasing control over their teaching practices, autonomy allows teachers to overcome external challenges and strengthens their commitment to innovation. Autonomy enhances confidence and reduces resistance, making it an essential condition for transforming innovation intentions into practice. Teachers in an autonomous environment are more likely to explore and gradually form stable innovation patterns. Therefore, teaching autonomy not only directly promotes teaching innovation but also strengthens the effect of AI literacy on the elements of the Theory of Planned Behavior, making psychological motivation more likely to turn into action. The following hypotheses are proposed:

*H5a. Teaching autonomy positively moderates the relationship between AI literacy and behavioral attitude.*

*H5b. Teaching autonomy positively moderates the relationship between AI literacy and subjective norm.*

*H5c. Teaching autonomy positively moderates the relationship between AI literacy and perceived behavioral control.*

### 3.4 Mediating role of the Theory of Planned Behavior

AI literacy not only directly affects teachers’ teaching innovation but also exerts an indirect effect through the three core components of the Theory of Planned Behavior: behavioral attitude, subjective norm, and perceived behavioral control (Ma, 2025). These three dimensions constitute the psychological mechanisms that enable teachers’ knowledge and skills to be transformed into visible innovative behaviors. In the behavioral attitude dimension, higher AI literacy helps teachers understand the value of AI in enhancing classroom efficiency, improving learning experiences, and achieving personalized support (Liu and Wang, 2024). Recognition of these values gradually accumulates into positive emotional experiences and solidifies into a positive attitude toward AI applications. Positive attitudes guide teachers to more readily experiment with tools, adjust processes, and conduct small-scale experiments in teaching practice, thus enhancing the continuity and scope of innovation activities. In the subjective norm dimension, AI literacy increases teachers’ sensitivity to the external environment (Adelana et al., 2024). Teachers can accurately interpret policy directions, peer practices, and student needs, perceiving widespread recognition of teaching innovation within the professional community. This recognition reinforces teachers’ social responsibility, transforming external pressure into self-identity, making innovation a natural choice for teaching. In the perceived behavioral control dimension, AI literacy strengthens teachers’ tool usage and problem-solving abilities, enhancing self-efficacy (Lim, 2023). Teachers believe they have the ability to deal with technical problems, classroom uncertainty, and resource shortages. Control enhances teachers’ confidence and stability when facing challenges, making innovative activities no longer high-risk trials, but sustainable routine practices. Attitude, subjective norm, and perceived behavioral control form the key psychological path through which AI literacy affects teaching innovation. These three factors work together, enabling teachers to move from having the capability to willingness to action, and ultimately to sustained innovation. The following hypotheses are proposed:

*H6a. Behavioral attitude mediates the relationship between AI literacy and teaching innovation.*

*H6b. Subjective norm mediates the relationship between AI literacy and teaching innovation.*

*H6c. Perceived behavioral control mediates the relationship between AI literacy and teaching innovation.*

**Figure 2:**
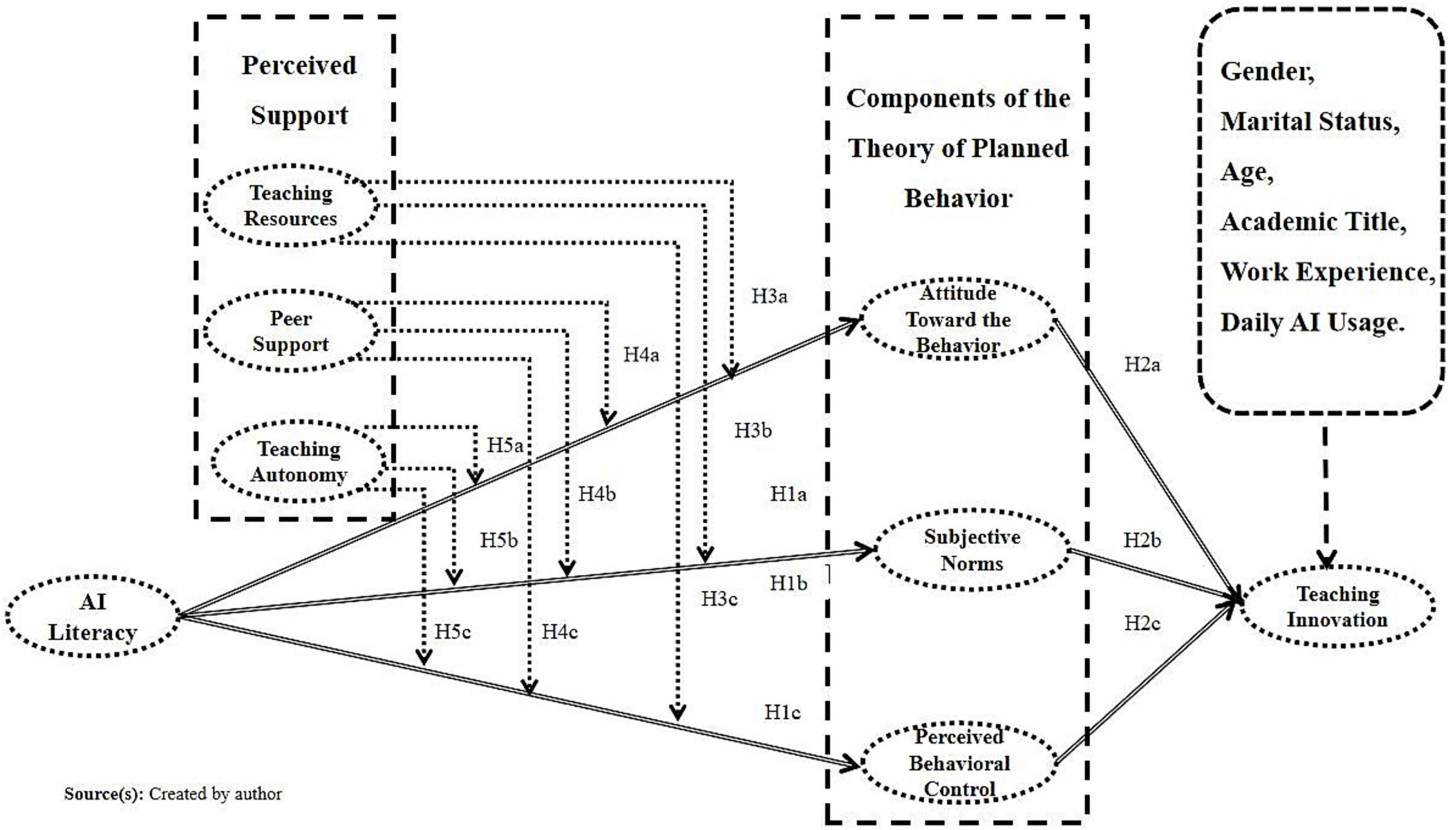
Research model.

In summary, the research model of this study is shown in Figure 2.

## 4 Method

### 4.1 Participants

The data for this study were collected online via the Wenjuanxing platform,^1^ using a seven-point Likert scale. To ensure sample relevance, two mandatory screening questions were placed at the start: (1) Occupational identity (only university teachers); (2) Experience using AI tools (must answer “Yes”). Respondents failing either screen were blocked from proceeding to the main questionnaire. Data cleaning followed a pre-specified process to ensure quality and consistency: (1) Duplicate response detection was performed, and surveys with duplicate responses from the same IP address or device were checked and excluded to avoid redundancy; (2) Response consistency was examined, and surveys where all items were answered with the same option were excluded; (3) Incomplete responses were removed. In total, 518 questionnaires were collected. After applying these criteria, 15 responses were excluded, resulting in 503 valid responses (effective response rate 97.1%). The valid sample represents a variety of universities across the country. The gender distribution was fairly balanced, with the majority of participants aged between 25 and 46 years. The sample included assistant professors, lecturers, associate professors, and professors, with years of experience ranging from 0–5 years to over 20 years. Most teachers reported using AI tools for more than 1 h daily, with some using them for over 5 h. Common platforms included ChatGPT, DeepSeek, Sora, and Wenxin Yiyan, indicating the widespread integration of AI in teaching and research practices. The demographic characteristics of the participants are shown in Table 3.

**Table 3:** Demographic characteristics.

Variable	Category	Frequency	Percentage
Gender	Male	274	54.47%
	Female	229	45.53%
Marital status	Married	349	69.38%
	Unmarried	154	30.62%
Age	25–35	215	42.74%
	36–46	156	31.01%
	47–57	96	19.09%
	58 and above	36	7.16%
Academic title	Teaching Assistant/No Title	194	38.57%
	Lecturer	178	35.39%
	Associate Professor	97	19.28%
	Professor	34	6.76%
Work experience	0–5 years	88	17.5%
	6–10 years	167	33.2%
	11–15 years	105	20.87%
	16–20 years	96	19.09%
	More than 21 years	47	9.34%
Daily AI usage	0–1 h	28	5.57%
	1–3 h	134	26.64%
	3–5 h	141	28.03%
	5–7 h	106	21.07%
	More than 7 h	94	18.69%

Source(s): created by author.

### 4.2 Measures

This study utilized established scales that have been empirically validated both domestically and internationally, with modifications made to suit the context of university teachers (Cui and Yin, 2023; Han et al., 2021; Liao et al., 2022; Liu et al., 2016; Ning et al., 2025; Richter and Schuessler, 2019; Wang et al., 2023; Zhang et al., 2025). All items were rated using a seven-point Likert scale (1 = strongly disagree, 7 = strongly agree), with higher scores indicating higher levels on each dimension. The measurement covered eight core variables: AI literacy (9 items, assessing teachers’ abilities to recognize, apply, evaluate AI tools, and their awareness of ethical risks), teaching innovation (5 items, reflecting practices such as exploring new ideas, applying diverse teaching methods, problem-solving, sharing experiences, and integrating resources), behavioral attitude (3 items, measuring teachers’ positive cognitive responses to organized teaching and research activities), subjective norm (3 items, reflecting social expectations from the department, colleagues, and academic groups), perceived behavioral control (3 items, addressing factors such as time, channels, and self-efficacy), teaching resources (3 items, evaluating institutional support for training, tools, and hardware facilities), peer support (3 items, reflecting experience sharing, encouragement, and collaboration among colleagues), and teaching autonomy (3 items, reflecting teachers’ freedom in decision-making related to the integration of AI in teaching). The specific items and scale sources for each variable are listed in Table 4.

**Table 4:** Measurement scales.

Variable	Item	Item description	Scale source
Artificial Intelligence Literacy (AIL)	AIL1	I can distinguish between AI-powered and non-AI-powered devices.	Ning et al. (2025), Wang et al. (2023)
	AIL2	I can identify AI technologies used in the applications or products I use daily.	
	AIL3	I understand how to apply AI tools to improve my teaching or research efficiency.	
	AIL4	I am proficient in using AI-related applications or products for teaching or research tasks.	
	AIL5	I can select the most appropriate AI tool or platform based on specific task requirements.	
	AIL6	I can assess the strengths and limitations of AI applications.	
	AIL7	When presented with multiple suggestions from an intelligent system, I can choose the most suitable solution.	
	AIL8	I actively consider ethical and privacy issues when using AI tools.	
	AIL9	I remain vigilant about the potential misuse of AI technologies in teaching or research.	
Teaching Innovation (TI)	TI1	I actively explore and experiment with new teaching concepts to enhance students’ cognitive engagement.	Cui and Yin (2023), Liu et al. (2016)
	TI2	I regularly apply diverse teaching methods or technologies in class to stimulate students’ interest in learning.	
	TI3	When faced with teaching challenges, I proactively adopt new strategies or approaches to solve problems.	
	TI4	I am willing to share my experiences of implementing new teaching ideas or methods with colleagues to receive feedback and support.	
	TI5	To achieve teaching innovation, I actively seek out and integrate necessary resources and tools (such as AI technologies, ICT, etc.).	
Behavior Attitude (BA)	BA1	I believe participating in organized research activities helps me gain more knowledge and academic resources in my field.	Liao et al. (2022), Zhang et al. (2025)
	BA2	I believe organized research activities improve my research efficiency.	
	BA3	I believe participating in organized research activities enhances the quantity and quality of my research output.	
Subjective Norm (SN)	SN1	My school or department encourages faculty to participate in organized research activities.	
	SN2	I believe my colleagues, mentors, or supervisors expect me to actively engage in organized research activities.	
	SN3	Many young faculty members around me are beginning to participate in organized research activities.	
Perceived Behavioral Control (PBC)	PBC1	I have sufficient time to participate in organized research activities.	
	PBC2	I am aware of the channels or platforms through which I can participate in organized research.	
	PBC3	I believe I have the necessary skills and experience to engage in organized research activities.	
Teaching Resources (TR)	TR1	My institution provides professional training and guidance on integrating AI technologies into teaching.	Han et al. (2021), Richter and Schuessler (2019)
	TR2	I have access to AI-related software and technological tools provided by my institution.	
	TR3	The school is equipped with basic hardware infrastructure that supports AI-integrated teaching (e.g., smart classrooms, teaching terminals).	
Peer Support (PR)	PR1	My colleagues are willing to offer advice and share their experiences on using AI in teaching.	
	PR2	When I encounter difficulties using AI technologies in teaching, I can receive encouragement and support from my colleagues.	
	PR3	My colleagues actively support and explore ways to integrate AI technologies into teaching.	
Teaching Autonomy (TA)	TA1	I have the autonomy to decide whether to integrate AI technologies into my teaching.	
	TA2	I can independently choose appropriate AI tools and methods based on my teaching goals.	
	TA3	I have the freedom to flexibly incorporate AI technologies throughout the teaching process.	

Source(s): created by author.

### 4.3 Common method Bias analysis

Since this study used self-reported questionnaires to collect data, there is a potential risk of common method bias (Podsakoff et al., 2003). To minimize this issue, several measures were implemented during the questionnaire design, including ensuring anonymity, adjusting the order of items, and incorporating attention check questions. In terms of statistical testing, Harman’s single-factor analysis was conducted. The results indicated that the first factor explained 28.213% of the variance, which is well below the 40% threshold, suggesting that a single factor did not dominate the data. Additionally, the variance inflation factors (VIFs) for the latent variables were all below 3.3 (Kock, 2015), further indicating that common method bias poses a limited threat to the study’s results.

## 5 Data analysis and results

This study employed Partial Least Squares Structural Equation Modeling (PLS-SEM) as the primary analytical method. PLS-SEM is suitable for complex models involving multiple latent variables and interaction effects, providing robust estimation results for both measurement and structural models (Henseler et al., 2015). PLS-SEM requires fewer assumptions regarding data distribution, making it particularly appropriate for exploratory and prediction-oriented research (Sarstedt et al., 2022). Compared to traditional covariance-based structural equation models, PLS-SEM offers greater flexibility and predictive power, especially in path analysis with multiple latent variables and indicators (Rigdon, 2016). Considering the inclusion of multiple core variables such as AI literacy, perceived support, teaching attitude, and teaching innovation, as well as the need to test their complex relationships, PLS-SEM aligns well with the research requirements and methodological approach (Carrión et al., 2017).

### 5.1 Measurement model evaluation

To assess the reliability and validity of the measurement tools, internal consistency and convergent validity of the latent variables were first analyzed (Table 5). The results showed that the Cronbach’s *α* values for all latent variables were above 0.80, and composite reliability (CR) exceeded 0.70, indicating strong internal consistency. Additionally, the standardized factor loadings for all measurement items were greater than 0.70 and significant, with average variance extracted (AVE) exceeding 0.50, meeting the recommended standards by Cheung et al. (2024), indicating good convergent validity for the constructs. In terms of discriminant validity, heterotrait-monotrait (HTMT) ratio analysis was conducted (Table 6). The results showed that the HTMT values for all variable pairs were below 0.85, consistent with the threshold set by Henseler et al. (2015), indicating good discriminant validity among the latent variables. Overall, the measurement model achieved high levels of reliability, convergent validity, and discriminant validity.

**Table 5:** Results of reliability and convergent validity testing.

Latent variable	Measurement items	Mean	Standard deviation	Factor loading	Cronbach’s α	CR	AVE
AIL	AIL1	4.507	1.539	0.773	0.920	0.932	0.606
	AIL2	4.513	1.572	0.792			
	AIL3	4.507	1.544	0.762			
	AIL4	4.473	1.554	0.787			
	AIL5	4.541	1.554	0.767			
	AIL6	4.489	1.529	0.782			
	AIL7	4.515	1.574	0.779			
	AIL8	4.513	1.520	0.788			
	AIL9	4.406	1.518	0.775			
TI	TI1	4.197	1.607	0.795	0.866	0.902	0.649
	TI2	4.181	1.601	0.793			
	TI3	4.241	1.594	0.806			
	TI4	4.203	1.510	0.811			
	TI5	4.215	1.663	0.823			
BA	BA1	4.320	1.679	0.863	0.840	0.904	0.758
	BA2	4.235	1.632	0.867			
	BA3	4.302	1.643	0.881			
SN	SN1	4.491	1.632	0.863	0.826	0.895	0.740
	SN2	4.489	1.621	0.858			
	SN3	4.491	1.619	0.859			
PBC	PBC1	4.433	1.651	0.871	0.836	0.901	0.752
	PBC2	4.408	1.689	0.852			
	PBC3	4.306	1.648	0.879			
TR	TR1	4.654	1.571	0.827			
	TR2	4.654	1.603	0.875			
	TR3	4.682	1.587	0.860			
PS	PS1	4.726	1.490	0.846	0.817	0.887	0.723
	PS2	4.791	1.474	0.824			
	PS3	4.805	1.603	0.880			
TA	TA1	4.718	1.690	0.879	0.838	0.900	0.750
	TA2	4.686	1.587	0.857			
	TA3	4.789	1.614	0.863			

Source(s): created by author.

**Table 6:** Results of discriminant validity testing.

Variable	AIL	BA	SN	PBC	TI	TR	PS	TA
AIL								
BA	0.362							
SN	0.405	0.506						
PBC	0.478	0.466	0.557					
TI	0.413	0.423	0.486	0.573				
TR	0.163	0.396	0.356	0.370	0.375			
PS	0.186	0.357	0.385	0.319	0.330	0.395		
TA	0.162	0.320	0.339	0.345	0.326	0.475	0.422	

Source(s): created by author.

### 5.2 Structural model analysis

To control for potential confounding effects, demographic variables such as gender, marital status, age, academic title, work experience, and AI usage duration were included as control variables (Cui and Yin, 2023; Jose et al., 2025; Zhao et al., 2022). The analysis indicated that these control variables did not have a significant impact on the main relationships, specifically the relationships between AI literacy, the components of the Theory of Planned Behavior (behavioral attitude, subjective norm, and perceived behavioral control), and teaching innovation. This suggests that the main relationships between AI literacy and teaching innovation are not influenced by these demographic factors, and the results are consistent across different population subgroups.

#### 5.2.1 Model explained variance and predictive relevance

Table 7 displays the explanatory power and predictive relevance of the structural model. The results indicated that the R^2^ values for the endogenous variables ranged from 0.269 to 0.309, indicating that the exogenous variables explained a substantial portion of the variance in the endogenous constructs. Additionally, the Stone-Geisser Q^2^ values for all endogenous variables were greater than zero (ranging from 0.190 to 0.224), suggesting strong robustness and reliability of the model in out-of-sample predictions. Overall, these results support the model’s rationality from both explanatory power and predictive relevance perspectives, further highlighting its theoretical value (Sarstedt et al., 2020; Shmueli et al., 2019).

**Table 7:** Explanatory power (R^2^) and predictive relevance (Q^2^) of the structural model.

Endogenous latent variables	*R* ^2^	Q^2^
BA	0.284	0.205
SN	0.269	0.190
PBC	0.309	0.224
TI	0.299	0.190

Source(s): created by author.

#### 5.2.2 Main effects path coefficient analysis and hypothesis testing

**Figure 3:**
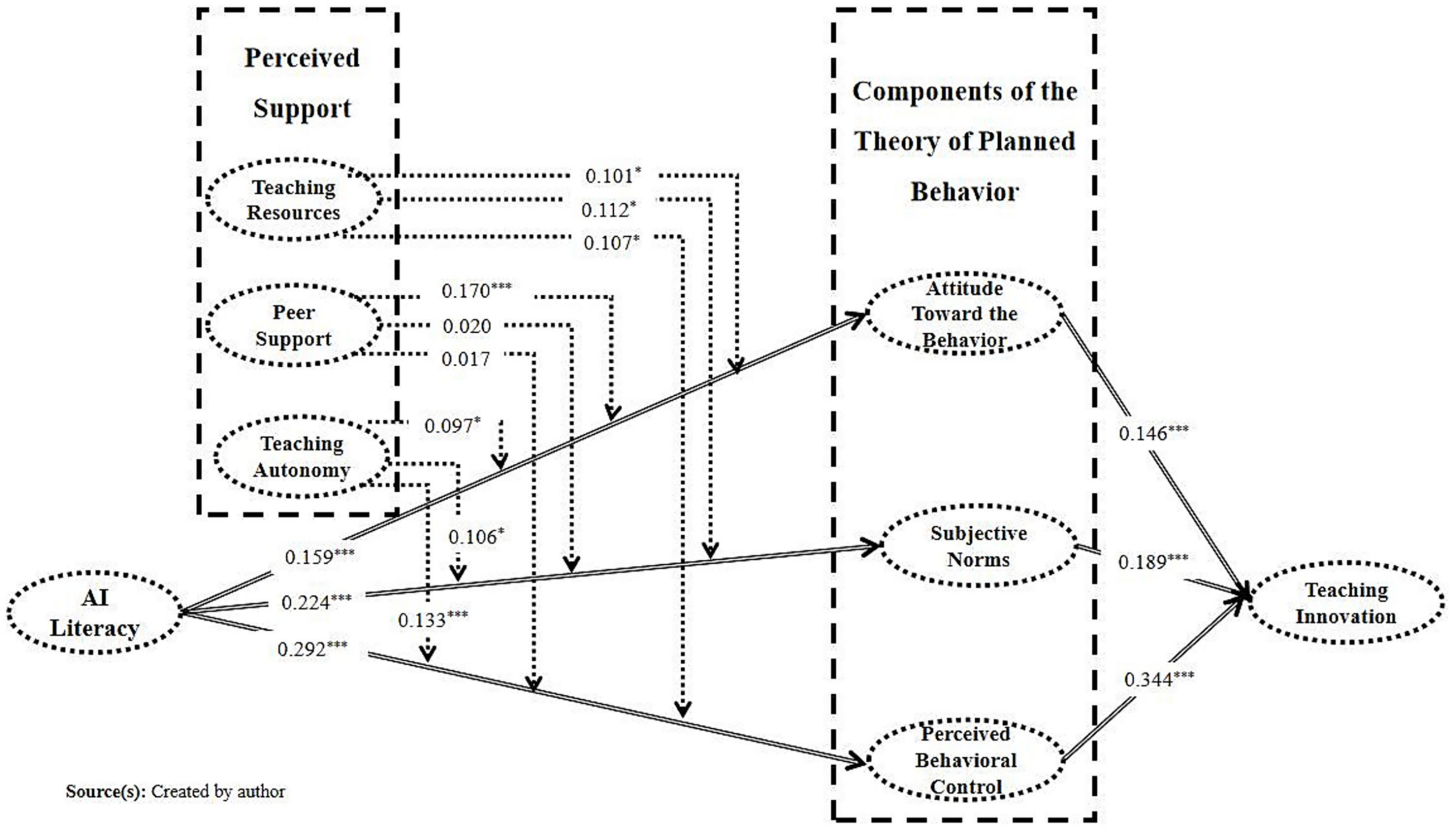
Structural model results.

Figure 3 presents the results of the main effects path coefficient tests for the structural model. AI literacy has a significant positive effect on teachers’ behavioral attitude (*β* = 0.159), subjective norm (*β* = 0.224), and perceived behavioral control (*β* = 0.292), supporting hypotheses H1a, H1b, and H1c. These results align with Ivanov et al. (2024), indicating that higher AI literacy helps teachers form positive teaching attitudes, enhances their perception of external normative expectations, and boosts their self-efficacy in applying AI in teaching. Furthermore, behavioral attitude (*β* = 0.146), subjective norm (*β* = 0.189), and perceived behavioral control (*β* = 0.344) all significantly predict teaching innovation, supporting hypotheses H2a, H2b, and H2c. Perceived behavioral control had the most significant effect, confirming Broadbent et al. (2024), Opoku et al. (2021)‘s finding that teachers are more likely to engage in innovative practices when they perceive greater control over teaching.

#### 5.2.3 Moderating effect analysis

Figure 3 illustrates the moderating effects of teaching resources, peer support, and teaching autonomy on the relationships between AI literacy and teachers’ behavioral attitude, subjective norm, and perceived behavioral control. Teaching resources showed significant positive moderating effects on all three paths. As the level of teaching resources increased, the influence of AI literacy on behavioral attitude ( β=0.101,p=0.043 ), subjective norm ( β=0.112,p=0.017 ), and perceived behavioral control ( β=0.107,p=0.021 ) strengthened, supporting hypotheses H3a, H3b, and H3c. This indicates that sufficient teaching resources provide perceived support for teachers and further enhance their positive attitudes and beliefs in the use of emerging technologies (Hazzan-Bishara et al., 2025).

Peer support had a significant moderating effect only on the relationship between AI literacy and behavioral attitude ( β=0.170,p=0.001 ), supporting hypothesis H4a. However, the effects on subjective norm ( β=0.020,p=0.653 ) and perceived behavioral control ( β=0.017,p=0.741 ) were not significant, and hypotheses H4b and H4c were not supported. This suggests that peer support is context-dependent in teachers’ technology adoption, more likely to influence the attitude dimension rather than universally affect all cognitive factors (Celik et al., 2025; Habibi et al., 2023).

Teaching autonomy exhibited significant positive moderating effects on all three paths. The higher the teaching autonomy, the stronger the impact of AI literacy on behavioral attitude ( β=0.097,p=0.036 ), subjective norm ( β=0.106,p=0.016 ), and perceived behavioral control ( β=0.133,p=0.004 ), supporting hypotheses H5a, H5b, and H5c. This result emphasizes the key role of teaching autonomy in fostering technology adoption and innovation practices, indicating that empowerment and decision-making autonomy effectively stimulate teachers’ proactivity and initiative in applying AI technologies (Bali et al., 2025; Hou and Shen, 2024).

**Figure 4:**
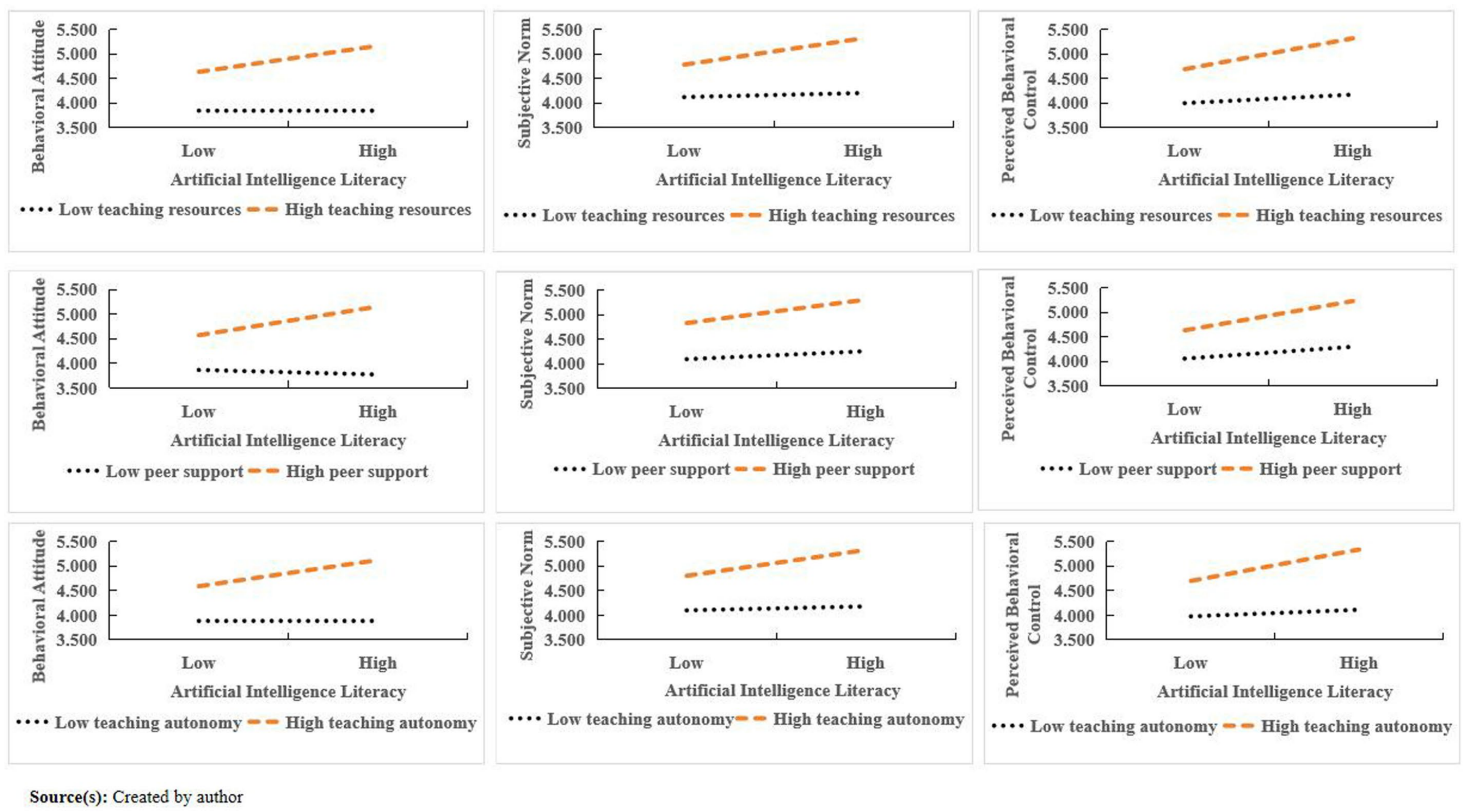
Simple slope plot.

Furthermore, to visually present the moderating effects, interaction effect plots for teaching resources, peer support, and teaching autonomy were generated (Figure 4), and the effects at different levels were reported (Table 8). The results showed that teaching resources had significant positive moderating effects on all three paths, with low-level effects being non-significant, medium-level effects enhancing, and high-level effects being the strongest (H3a–H3c). Peer support had significant effects only on the behavioral attitude path (H4a), with no significant effects on subjective norm or perceived behavioral control (H4b and H4c). Teaching autonomy exhibited significant positive moderating effects on all three paths, and the effects strengthened as the level of autonomy increased (H5a–H5c).

**Table 8:** Moderating effect at different levels.

Independent variable	Dependent variable	Moderator variable	TA	β	95%CI		*p*-value
AIL	BA	TR	Low	−0.008	−0.165	0.149	0.923
			Medium	0.262	0.165	0.359	0.000^***^
			High	0.531	0.412	0.650	0.000^***^
	SN		Low	0.083	−0.071	0.236	0.291
			Medium	0.310	0.215	0.405	0.000^***^
			High	0.538	0.421	0.654	0.000^***^
	PBC		Low	0.174	0.021	0.328	0.026^*^
			Medium	0.401	0.306	0.496	0.000^***^
			High	0.628	0.511	0.744	0.000^***^
	BA	PS	Low	−0.093	−0.260	0.073	0.270
			Medium	0.245	0.147	0.343	0.000^***^
			High	0.584	0.460	0.708	0.000^***^
	SN		Low	0.162	−0.001	0.325	0.052
			Medium	0.320	0.224	0.416	0.000^***^
			High	0.478	0.356	0.600	0.000^***^
	PBC		Low	0.241	0.076	0.407	0.004^**^
			Medium	0.418	0.320	0.516	0.000^***^
			High	0.595	0.471	0.719	0.000^***^
	BA	TA	Low	−0.014	−0.179	0.150	0.863
			Medium	0.259	0.159	0.359	0.000^***^
			High	0.533	0.413	0.652	0.000^***^
	SN		Low	0.081	−0.078	0.240	0.318
			Medium	0.304	0.207	0.400	0.000^***^
			High	0.527	0.411	0.642	0.000^***^
	PBC		Low	0.135	−0.023	0.293	0.094
			Medium	0.387	0.291	0.483	0.000^***^
			High	0.639	0.524	0.753	0.000^***^

*p < 0.05; **p < 0.01; ***p < 0.001.

Source(s): created by author.

#### 5.2.4 Mediation effect analysis

Using SmartPLS, the mediation effects of behavioral attitude (BA), subjective norm (SN), and perceived behavioral control (PBC) in the relationship between AI literacy (AIL) and teaching innovation (TI) were tested based on 5,000 bootstrap samples. According to Hayes (2009), indirect effects are considered significant if the 95% confidence interval does not include zero. The results (Table 9) indicated that all three mediation paths were significant, with no confidence intervals crossing zero. Specifically, the indirect effect of AI literacy through BA was relatively small ( β=0.023,p<0.001 ), supporting H6a; the effect through SN was moderate ( β=0.042,p<0.001 ), supporting H6b; and the effect through PBC was the largest ( β=0.101,p<0.001 ), supporting H6c. Additionally, the direct effect of AI literacy on TI remained significant ( β=0.166,p<0.001 ), indicating that BA, SN, and PBC partially mediate the relationship between AI literacy and teaching innovation. These findings confirm Ramnarain et al. (2024), suggesting that AI literacy not only directly enhances teachers’ innovation inclination but also indirectly boosts innovation momentum through multiple psychological mechanisms (attitudes, norms, control beliefs).

**Table 9:** Results of mediating effect analysis.

Mediating path	Effect value	S. E	Lower 2.5%	Upper 2.5%	*p*
AIL→BA→TI	0.023	0.010	0.007	0.045	***
AIL→SN → TI	0.042	0.013	0.020	0.070	***
AIL→PBC → TI	0.101	0.018	0.066	0.138	***
AIL→TI	0.166	0.022	0.125	0.211	***

***p < 0.001.

Source(s): created by author.

## 6 Discussion

AI literacy significantly enhances teachers’ behavioral attitude, subjective norm, and perceived behavioral control (H1a–H1c are supported). The results suggest that AI literacy is not merely a technical skill but also a cognitive and psychological resource. Higher AI literacy helps teachers deepen their understanding of the educational value of AI, fostering the formation of a positive attitude (Ivanov et al., 2024; Ji et al., 2025). As a multidimensional construct, AI Literacy shapes teachers’ psychological and cognitive readiness to embrace new technologies. By understanding AI’s potential in education, teachers are more likely to develop positive attitudes toward its use, which, in turn, enhances their willingness to engage in innovative teaching practices. The strengthening of subjective norm indicates that teachers with higher literacy are more likely to recognize and internalize the expectations from external sources, such as policies, colleagues, and students, which further reinforces their professional responsibilities (Dierendonck et al., 2024). Aligns with TPB (Ajzen, 1991), these findings highlight the role of external expectations in shaping behavior. Teachers with higher AI literacy are not only more attuned to these external norms but are also more likely to integrate them into their professional identity, driving their engagement with AI in teaching. This emphasizes the significant role of external pressures and institutional support in facilitating teaching innovation. The enhancement of perceived behavioral control shows that AI literacy boosts self-efficacy, enabling teachers to navigate challenges and use AI tools in their teaching practices, thereby creating a mechanism of “technological mastery—efficacy improvement—behavioral transformation” (Viberg et al., 2024). Such findings underline the critical role of self-efficacy in fostering teaching innovation. When teachers feel competent and confident in using AI tools, they are more likely to engage in experimental and innovative behaviors, breaking free from traditional teaching models. This supports Wang and Zhao (2021), who emphasize the central role of self-efficacy in translating knowledge and skills into actual behaviors.

Behavioral attitude, subjective norm, and perceived behavioral control all significantly and positively predict teaching innovation (H2a–H2c are supported). This finding further validates the importance of these three psychological factors in the Theory of Planned Behavior (TPB) for translating intentions into actual behaviors. Perceived behavioral control had the most significant effect, indicating that when teachers feel confident in mastering and using AI, they are more likely to move away from traditional models and experiment with new practices. Specifically, when teachers feel equipped with the necessary skills and confidence to handle challenges, they are more likely to break free from conventional methods and engage in innovative behaviors. This aligns with the findings of Opoku et al. (2021) and Ramnarain et al. (2024), confirming the central role of self-efficacy in teaching innovation. A positive behavioral attitude reflects the recognition of AI’s educational value, which in turn translates into motivation for innovation. Teachers, who understand the potential of AI in education, are more inclined to incorporate AI into their teaching practices. Granström and Oppi (2025) emphasize that teachers’ positive attitudes are not just about technical proficiency but also about the recognition of AI’s broader educational value, which drives them to innovate. The formation of such an attitude is underpinned by a shift from mere technical acceptance to a deeper understanding of the educational benefits, providing teachers with the motivation needed to embrace innovation. The impact of subjective norms shows that when teachers feel external expectations, they perceive innovation as an essential way to fulfill their professional roles and responsibilities. Policy support, peer expectations, and student demands play key roles in driving teachers’ engagement with innovation (Cai and Tang, 2022). This finding highlights the significant influence of external pressures on shaping teachers’ behavior. Teachers not only internalize these external expectations but also integrate them into their professional identity, reinforcing their commitment to adopting AI in their teaching practices. This underscores the role of institutional support and societal norms in facilitating teaching innovation.

Mediation analysis reveals that behavioral attitude, subjective norm, and perceived behavioral control all partially mediate the relationship between AI literacy and teaching innovation (H6a–H6c are supported). Among these, perceived behavioral control emerged as the most significant mediator, emphasizing the central role of self-efficacy in translating AI literacy into practical teaching innovation. This finding underscores the idea that teachers who feel confident in their ability to use AI tools are more likely to engage in innovative behaviors. As noted by Wang and Zhao (2021), self-efficacy plays a critical role in bridging the gap between knowledge acquisition and actual behavioral change, making it a key factor for fostering teaching innovation. While behavioral attitude and subjective norm also mediate the relationship, their effects were comparatively weaker. Behavioral attitude, which reflects teachers’ recognition of AI’s educational value, plays an important role in motivating innovation. However, without sufficient confidence in AI’s practical application, sustaining innovation becomes challenging. As Peng et al. (2024) argue, positive attitudes alone are not enough to overcome barriers to adoption. Teachers must feel equipped with the necessary skills and support to translate their recognition of AI’s value into consistent and meaningful teaching practices. The subjective norm, which relates to external expectations from peers, policies, and institutional pressures, can also play a role in promoting innovation. However, its mediating effect is more limited. Relying solely on external pressure can lead to compliance-based innovation, where teachers adopt new methods only because they feel obligated rather than motivated by a genuine desire for exploration and growth (Lu and Wang, 2023). Such innovations are more likely to be superficial and short-lived, as they lack intrinsic motivation or autonomy.

Moderating effect analysis shows that perceived support conditions play an important role in the “AI literacy—psychological mechanism” path. Teaching resources exhibited significant positive moderating effects on all three paths (H3a–H3c are supported). The availability of resources provides teachers with necessary tools, technical training, and institutional support, making it easier for AI literacy to translate into positive attitudes, norms, and control beliefs (Hazzan-Bishara et al., 2025). The effect of peer support was selective, being significant only in the relationship between AI literacy and behavioral attitude (H4a is supported), with no significant effects on subjective norm and perceived behavioral control (H4b and H4c are not supported). Attitude formation relies on emotional resonance and value recognition, with peers providing psychological support through experience sharing and belief dissemination (Habibi et al., 2023). Subjective norms are more shaped by policy guidance and institutional requirements, and informal peer opinions are less likely to serve as primary reference points (Dierendonck et al., 2024). Perceived behavioral control relies on teachers’ self-confirmation of their abilities, with efficacy developed through accumulated experience, technical mastery, and teaching feedback, thus limiting the role of peer support (Gordon et al., 2023). University teachers typically have high professional autonomy, relying more on institutional signals and personal experience than on peer opinions for normative cognition and ability judgment. Therefore, the influence of peer support is concentrated in the attitude dimension, with boundaries in the formation of normative cognition and efficacy. Teaching autonomy exhibited significant positive moderating effects on all three paths (H5a–H5c are supported). In high-autonomy environments, teachers have greater decision-making power and freedom to experiment, allowing them to flexibly integrate AI tools in teaching design and practice. Self-determination theory (Deci and Ryan, 2000) suggests that autonomy can stimulate intrinsic motivation and exploratory desire, while Hou and Shen (2024) further emphasize its role in promoting responsibility and sustainability. The study results confirm the critical role of teaching autonomy in transforming AI literacy into innovative behavior.

## 7 Contributions, limitations, and future research directions

### 7.1 Theoretical contributions

This study develops a framework based on the Theory of Planned Behavior (TPB) to explore how AI literacy influences teaching innovation through psychological mechanisms such as behavioral attitude, subjective norm, and perceived behavioral control. The research also introduces perceived support factors such as teaching resources, peer support, and teaching autonomy, further revealing their moderating roles between AI literacy and teaching innovation. The theoretical contributions of this study are as follows:

First, it expands the psychological connotations of AI literacy. Existing studies often regard AI literacy as an external manifestation of technical abilities and tool usage (Ng et al., 2021), while this research redefines AI literacy from a psychological perspective. The study finds that AI literacy is not just a technical competence but also a cognitive and psychological resource that influences teachers’ behavioral attitude, subjective norm, and perceived behavioral control. Teachers with higher AI literacy are more likely to recognize the potential of AI in education, forming a positive teaching attitude. This finding provides a new definition for the theoretical system of AI literacy and reveals its multidimensional impact on teachers’ psychology and behavior (Ayanwale et al., 2024; Sperling et al., 2024).

Second, it deepens the application of TPB in educational technology adoption. This study enriches the application of TPB in the educational field by verifying the roles of behavioral attitude, subjective norm, and perceived behavioral control in teaching innovation. It reveals how AI literacy influences teachers’ innovative behaviors through these three dimensions. Specifically, the study demonstrates that perceived behavioral control (self-efficacy) plays a central role in teachers’ innovative behaviors. AI literacy enhances self-efficacy, helping teachers overcome technological challenges and promote teaching innovation (Frawley and Campbell, 2025; Gold et al., 2024). This finding not only deepens the theoretical application of TPB in educational technology adoption but also provides a new theoretical framework for understanding teachers’ innovative behaviors (Bali et al., 2025).

Third, it fills the gap in existing theories by incorporating the Theory of Planned Behavior into the study of teaching innovation. Previous theories on technology adoption and teaching innovation often overlook the psychological mechanisms that drive teachers’ willingness to adopt new tools and engage in innovative behaviors. For instance, traditional models have often failed to explicitly account for the role of subjective norms and perceived behavioral control in influencing teachers’ innovative actions. By integrating these components of TPB, this study provides a more comprehensive understanding of how teachers’ psychological states—shaped by both internal capabilities (e.g., attitudes) and external expectations (e.g., norms)—interact to influence their engagement in innovation (Ma and Lei, 2024). TPB’s inclusion of both cognitive and social dimensions of decision-making offers a more robust theoretical framework for analyzing educational innovation.

Finally, it explores the moderating role of perceived support factors in teaching innovation. By incorporating teaching resources, peer support, and teaching autonomy into the TPB framework, this study investigates their moderating effects between AI literacy and teaching innovation. The results show that adequate teaching resources, effective peer support, and higher teaching autonomy significantly enhance the impact of AI literacy on teachers’ psychological mechanisms, further promoting teaching innovation (Ramnarain et al., 2024). This finding provides new insights into the internal and external drivers of teachers’ innovative behaviors, expands the application boundaries of TPB, and offers theoretical support for future educational policies and teacher training designs (Cao et al., 2022).

### 7.2 Practical implications

This study investigates the mechanisms through which university teachers’ AI literacy influences teaching innovation, providing several implications for educational practice and policy-making.

First, teacher training should go beyond basic technical operation and redefine AI literacy as a comprehensive capability encompassing both cognitive and psychological aspects. Universities can design modular courses and practical seminars to help teachers master the application of tools, while reinforcing educational value recognition and critical thinking through case analysis and scenario exercises, thus enhancing overall literacy on both skills and psychological levels.

Second, teaching innovation depends on the enhancement of teachers’ perceived behavioral control. Administrators should foster self-efficacy through continuous feedback, progressive tasks, and simulated teaching, allowing teachers to maintain confidence in uncertain and challenging situations. Higher levels of perceived control can translate into stable innovative intentions and practices.

Third, perceived support is a crucial condition for fostering teachers’ innovation. Schools must ensure the availability of educational resources, including technical training, digital platforms, and interdisciplinary collaboration opportunities, to strengthen teaching preparation. Peer support can be implemented through academic community building, experience sharing, and collaborative projects, providing emotional support on the value and affective levels. Teaching autonomy should be guaranteed through institutional arrangements, empowering teachers with decision-making authority in course design, tool selection, and teaching methods, thereby stimulating exploration motivation and continuous innovation.

Finally, education policymakers should consider multilevel needs. National and regional policies should incentivize the balanced distribution of AI education resources; at the school level, layered training should be designed based on teachers’ professional development stages; at the individual level, flexible autonomy and continuous support should be provided to guide teachers in transforming AI literacy into visible teaching innovation practices.

### 7.3 Research limitations

The data in this study were sourced from university teachers in China, and the sample is concentrated in terms of geographic and institutional backgrounds, limiting the generalizability of the findings across different cultures and institutional contexts. The research design used a cross-sectional survey, which makes it difficult to fully validate causal relationships between variables. The research tool mainly relied on self-reported questionnaires, which may have introduced social desirability bias and subjective bias. The dimensions of perceived support factors were relatively limited, focusing only on teaching resources, peer support, and teaching autonomy, without addressing broader contextual variables such as leadership support, organizational climate, and educational policies. The model also lacked a thorough examination of differences among teacher groups, with insufficient exploration of heterogeneity across disciplines and career stages. The research methodology predominantly used quantitative analysis, leaving limited space for capturing teachers’ real psychological experiences and practical logics.

### 7.4 Future research directions

Future research could expand the sample to include university teachers from different countries and regions to test the universality and differences of AI literacy across diverse cultural and institutional contexts. Longitudinal tracking and experimental designs could be employed to observe the development of teachers’ literacy and innovation pathways over time. The research dimensions of perceived support should be extended to include leadership support, organizational climate, and educational policies, constructing a more comprehensive contextual framework. Future studies could also focus on the heterogeneity of teacher groups, comparing differences in the mechanisms across disciplines and career stages, and revealing the interactive effects between group and individual factors. The research methodology could combine both qualitative and quantitative approaches, using interviews, classroom observations, and case studies to gain a deeper understanding of teachers’ psychological experiences and practical logics in teaching innovation.

## Data Availability

The raw data supporting the conclusions of this article will be made available by the authors, without undue reservation.

## Footnotes

^1^ https://www.wjx.cn/vm/Y8VN9Xp.aspx#
